# IL-1β Inhibition in Cardiovascular Complications Associated to Diabetes Mellitus

**DOI:** 10.3389/fphar.2017.00363

**Published:** 2017-06-13

**Authors:** Concepción Peiró, Óscar Lorenzo, Raffaele Carraro, Carlos F. Sánchez-Ferrer

**Affiliations:** ^1^Department of Pharmacology, School of Medicine, Universidad Autónoma de MadridMadrid, Spain; ^2^Instituto de Investigación Sanitaria Hospital Universitario de La Paz (IdiPAZ)Madrid, Spain; ^3^Department of Medicine, School of Medicine, Universidad Autónoma de MadridMadrid, Spain; ^4^Instituto de Investigación Sanitaria Fundación Jiménez DíazMadrid, Spain; ^5^Service of Endocrinology, Hospital de La PrincesaMadrid, Spain; ^6^Instituto de Investigación Sanitaria Hospital de La PrincesaMadrid, Spain

**Keywords:** diabetes, inflammation, cardiovascular complications, interleukin-1β, interleukin-1 inhibitors

## Abstract

Diabetes mellitus (DM) is a chronic disease that affects nowadays millions of people worldwide. In adults, type 2 diabetes mellitus (T2DM) accounts for the majority of all diagnosed cases of diabetes. The course of the T2DM is characterized by insulin resistance and a progressive loss of β-cell mass. DM is associated with a number of related complications, among which cardiovascular complications and atherosclerosis are the main cause of morbidity and mortality in patients suffering from the disease. DM is acknowledged as a low-grade chronic inflammatory state characterized by the over-secretion of pro-inflammatory cytokines, including interleukin (IL)-1β, which reinforce inflammatory signals thus contributing to the development of complications. In this context, the pharmacological approaches to treat diabetes should not only correct hyperglycaemia, but also attenuate inflammation and prevent the development of metabolic and cardiovascular complications. Over the last years, novel biological drugs have been developed to antagonize the pathophysiological actions of IL-1β. The drugs currently used in clinical practice are anakinra, a recombinant form of the naturally occurring IL-1 receptor antagonist, the soluble decoy receptor rilonacept and the monoclonal antibodies canakinumab and gevokizumab. This review will summarize the main experimental and clinical findings obtained with pharmacological IL-1β inhibitors in the context of the cardiovascular complications of DM, and discuss the perspectives of IL-1β inhibitors as novel therapeutic tools for treating these patients.

## Diabetes, Inflammation and Cardiovascular Complications

Diabetes mellitus (DM) is a cardiometabolic disease that affects millions of people worldwide. Accordingly, the World Health Organization (WHO) has acknowledged the disease as epidemic. In adults, the prevalence of type 2 diabetes mellitus (T2DM) is markedly higher that that of type 1 diabetes mellitus (T1DM), since it accounts for at least 90% of all diagnosed cases of diabetes. The rapid increase in the prevalence of T2DM has emerged as a major global health problem, mostly due to associated complications, medical costs and reduced life expectancy. The course of the T2DM is characterized by insulin resistance and a progressive loss of β-cell mass, together with the onset of vascular complications including coronary heart disease, stroke, peripheral vascular disease, and end stage renal disease, making it a leading cause of death worldwide.

Diabetes mellitus is nowadays acknowledged as a low-grade chronic inflammatory condition characterized by the over-secretion of pro-inflammatory cytokines. A growing body of evidence currently points at interleukin-1β (IL-1β), which is a major player in a wide array of auto-inflammatory diseases, to also act as key promoter of systemic and tissue inflammation in DM (Dinarello et al., 2010; Sumpter et al., 2011). Indeed, an enhanced expression of IL-1β in a high glucose milieu has been described in human monocytes and macrophages (Shashkin et al., 2006; Dasu et al., 2007), pancreatic islets (Maedler et al., 2004), myocardium (Niu et al., 2014), and aortic endothelium (Asakawa et al., 1997), while the upregulated IL-1β levels have been described in the heart and the retina and retinal vessels from diabetic rats, among other (Ares-Carrasco et al., 2009; Liu et al., 2012).

Many pieces of evidence, including studies in humans, suggest that IL-1β plays a role in insulin resistance, both in clinically overt T2DM and pre-diabetic states (van Asseldonk et al., 2011). Such conditions are characterized by an over-production of adipocytokines, including IL-1β, that are locally associated to the inflammation of the adipose tissue. Importantly, these adipokines can also be released to the circulation and impact on distant organs, including the heart or the vessels. In fact, increased systemic and vascular inflammation are considered key mechanisms underlying diabetic vasculopathy (Sowers, 2013). In this context, a key role of pro-inflammatory cytokines, such as IL-1β, in the development of cardiovascular complications of DM is being considered (Raines and Ferri, 2005; Goldberg, 2009; Sprague and Khalil, 2009; Frostegård, 2013; Krishnan et al., 2014).

Based on the above described autoinflammatory features of DM, the pharmacological approaches to treat diabetes should not only correct hyperglycaemia, but also target chronic inflammation in order to prevent the development of metabolic and cardiovascular complications. In this context, blocking IL-1β arises as a challenging therapeutic option to treat the progression of the disease and its complications.

## Il-1β and the Inflammasome

Interleukins are regulatory proteins with ability to accelerate or inhibit inflammatory processes, as well as other tissue responses. IL-1 belongs to the group of pro-inflammatory interleukins together with IL-2, IL-6, IL-7, IL-8, IL-15, IL-17, and IL-18. IL-1 may counterregulate anti-inflammatory cytokines such as IL-4, IL-10, IL-11, or other cytokines, such as IL-12 and IL-13 (Fisman et al., 2008). The IL-1 superfamily of cytokines comprises IL-1α, IL-1β, the endogenous regulator of IL-1 activity [a competitive IL-1 receptor antagonist (IL-1Ra)], IL-18, and the newly discovered IL-33, among other. The IL-1 superfamily is closely linked to both innate inflammation and immune responses. IL-1α is constitutively expressed in most cells of healthy subjects, although a role for this cytokine in disease is being validated in the context of sterile inflammation (Dinarello et al., 2012). In contrast, the expression of IL-1β is very limited in health, but it is markedly enhanced in blood monocytes, tissue macrophages and dendritic cells after stimuli like microbial products and other cytokines, such as tumor necrosis factor (TNF)α, IL-18, IL-1α, or IL-1β itself, as an autoinflammatory mechanism (Dinarello et al., 2012).

Interleukin-1β is produced as a precursor peptide (pro-IL-1β), which is N-terminal cleaved by caspase-1 or by the IL-1 converting enzyme (ICE) to form the active mature molecule. Caspase-1, an intracellular cysteine protease, needs firstly to be processed following the oligomerization of a complex of intracellular proteins termed the inflammasome. A key component of the inflammasome, NLRP3, plays a critical role in the secretion of IL-1β and in pyroptosis, which is an inherently inflammatory caspase 1-dependent mechanism of cell death triggered by various pathological stimuli, such as acute myocardial infarction (AMI) (Bergsbaken et al., 2009). Remarkably, a human autosomal mutation in NLRP3 results in enhanced caspase-1 activity and greater secretion of IL-1β (Franchi et al., 2009).

Interleukin-1β triggers intracellular signaling cascades through the activation of the interleukin-1 receptor, type I (IL-1R1), which also binds IL-1α. IL-1R1 is characterized by extracellular immunoglobulin-like domains and an intracellular Toll/interleukin-1R (TIR) domain, and it requires heterodimer formation with interleukin-1 receptor accessory protein (IL-1RAcP) to exert intracellular signaling (Palomo et al., 2015). IL-1R1 is expressed in all cells and its activation triggers multiple and sequential phosphorylation events that result in nuclear translocation of transcription factors. IL-1 activates JAK protein kinases that phosphorylate serine and threonine residues, which are the targets of the mitogen-activated protein kinase (MAPK) family. MAPKs then stimulate the translocation of nuclear factor (NF)-κB to the nucleus via the IRAK-TRAF6 pathway, and enhance nuclear binding of c-jun and c-fos, for activator protein (AP)-1 activation. Both NF-κB and AP-1 sites are present in the promoter regions of many inflammation-related IL-1-inducible genes that encode for diverse cytokines, adhesion molecules, chemokines or pro-inflammatory enzymes, among other (Palomo et al., 2015).

## Pharmacological Blockade of Il-1β

In view of the growing pathophysiological relevance of IL-1β in a wide variety of diseases, novel biological drugs have been developed over the last years to antagonize the actions of the cytokine. The drugs targeting IL-1β that are currently approved for clinical use are anakinra, rilonacept, and canakinumab, while gevokizumab has received orphan drug designation. A brief description of these drugs follows below.

Anakinra is a short-acting recombinant non-glycosylated form of the naturally occurring IL-1Ra, which blocks the activity of both IL-1α and IL-1β. Anakinra was first approved for the treatment of rheumatoid arthritis on a daily basis in 2001 (Dinarello et al., 2012). However, its clinical indications have been extended so far to other diseases such as the cryopyrin-associated periodic syndromes (CAPS), a group of rare inherited auto-inflammatory diseases associated to pathogenic variants in the IL-1-regulating genes NLRP3 and ILRN (Jesus and Goldbach-Mansky, 2014). Anakinra nowadays occupies a relevant position in IL-1 therapeutics as a result, of its excellent safety record even in long-term treatments, among other (Kullenberg et al., 2016).

The development of soluble decoy receptors was later introduced as a strategy to achieve IL-1 binding and neutralization. Rilonacept is a long-acting dimeric fusion protein that complexes the extracellular residues of the two IL1 receptor subunits, IL-1R1 and IL-1RAcP, to the Fc portion of IgG1. Rilonacept was approved in 2008 by the FDA for the treatment of CAPS. Like anakinra, rilonacept binds both IL-1α and IL-1β.

More recently, monoclonal antibodies that specifically target IL-1β have been developed. Canakinumab is a human IgG1k monoclonal antibody that binds and neutralizes soluble IL-1β, with no cross-reactivity with other interleukins, including IL-1α. Like rilonacept, canakinumab was approved for treating CAPS, other rare periodic fever syndromes, and juvenile arthritis (Dinarello et al., 2012). A second humanized IgG2 monoclonal antibody, gevokizumab, which also strongly binds IL-1β has been designed as orphan drug by the Food and Drug Administration (FDA) and the European Medicine Agency (EMA) for treating a series of rare conditions, including pyoderma gangrenosum, Schnitzler syndrome, chronic non-infectious uveitis or congenital hyperinsulinism. Another humanized IL-1 neutralizing antibody LY2189102 (Eli-Lilly and Company) is currently under study (Sloan-Lancaster et al., 2013).

Furthermore, a novel approach to block IL-1β has been developed by means of active vaccination against endogenous pro-inflammatory proteins. The vaccine hIL1bQb consists of full-length recombinant IL-1β coupled to virus-like particles, and it has been shown to produce endogenous anti-IL-1β antibodies in pre-clinical models. The vaccine is currently starting to being tested in patients (Cavelti-Weder et al., 2016).

Beyond their yet approved indications, these drugs are being currently used in both pre-clinical research and clinical trials to assess their applicability to other conditions in which IL-1β may play a key pathophysiological role. In the next sections, the main pre-clinical findings on the modulation of cardiovascular dysfunction by IL-1β pharmacological blockers will be summarized. Moreover, the clinical trials assessing the cardiovascular impact of IL-1β inhibition in the context of DM will be reviewed.

## Il-1β Blockade and Experimental Diabetic Vasculopathy

### Endothelial Dysfunction

Endothelial dysfunction is a crucial and early manifestation of vascular diseases. It is characterized by the impairment of the relaxations induced by nitric oxide (NO) and other vasodilator compounds (like prostacyclin or the endothelium hyperpolarizing factor), while the endothelial vasoconstrictor factors are increased (Félétou and Vanhoutte, 2006). A pro-oxidant and pro-inflammatory vascular environment is another characteristic of endothelial dysfunction (Félétou and Vanhoutte, 2006). Endothelial dysfunction is linked to the early development of DM in both humans and experimental animal models, and associated to reactive oxygen species (ROS) over-production (Durante et al., 1988; Calver et al., 1992; Johnstone et al., 1993; Angulo et al., 1998; Rodríguez-Mañas et al., 2003a,b).

There is also increasing evidence suggesting that the development of an inflammatory environment in the vasculature by pro-inflammatory cytokines is followed by an impairment of endothelial function. Thus, acute systemic inflammation in response to *Salmonella typhi* vaccine produces a temporary but profound dysfunction of human arterial endothelium in both resistance and conduit vessels, which is related to cytokine production (Hingorani et al., 2000; Kharbanda et al., 2002). Moreover, it is now well established, either in experimental models and humans, that pro-inflammatory cytokines impair vascular reactivity in different vascular beds, including resistance vessels (Vila and Salaices, 2005).

Although the IL-1 pathway is considered at present a critical player in the pathophysiology of both T1DM and T2DM (Donath and Shoelson, 2011; Herder et al., 2015), the evidence on the impact of IL-1β on vascular function is still limited. In isolated resistance microvessels from non-diabetic animals, IL-1β produces endothelial dysfunction after different exposure times to the cytokine (Wimalasundera et al., 2003; Jiménez-Altayó et al., 2006; Vallejo et al., 2014). Interestingly, the impairment of endothelial function may occur even after a rather short incubation with IL-1β (30 to 120 min), when the possible pro-inflammatory responses triggered by the cytokine are not fully developed, as indicated by the lack of involvement of inducible pro-inflammatory enzymes that require *de novo* synthesis (Vallejo et al., 2014). This early endothelial dysfunction evoked by IL-1β is rather due to the IL-1 receptor-mediated activation of NADPH oxidase, which enhances superoxide anion ($O_{2}^{•–}$) production (Vallejo et al., 2014). Interestingly, NADPH oxidase over-activation has been linked to excess ROS generation and the development of atherosclerosis in the context of diabetic vasculopathy (Olukman et al., 2010; Gray et al., 2013). The pharmacological blockade of IL-1 receptors by anakinra permits to attenuate both NADPH activation and endothelial dysfunction induced by IL-1β in microvessels from non-diabetic animals (Vallejo et al., 2014).

Interestingly, the intraperitoneal administration of anakinra can also partially recover the endothelial dysfunction observed in experimental diabetes after 15 days of diabetes induction (Vallejo et al., 2014). The animals receiving the drug exhibited a clear improvement of endothelium-dependent relaxations that was paralleled by the normalization of NADPH oxidase activity in the vascular wall (Vallejo et al., 2014). Intriguingly, the circulating levels of IL-1β were not found to be enhanced (Vallejo et al., 2014), as previously reported by others in experimental models (Yazar et al., 2011) or in diabetic patients (Mooradian et al., 1991; Pereira et al., 2014). The local over-expression of IL-1β in the vascular wall may be responsible for a paracrine inflammatory response, similarly to that described in vitreous samples from patients with proliferative diabetic retinopathy (Patel et al., 2008). In addition, other long-lasting pro-inflammatory mechanisms induced by IL-1β can be involved in the endothelial dysfunction associated to experimental diabetes, since improved endothelium-dependent relaxation was observed after the pharmacological blockade of cyclooxygenase or the inducible form of nitric oxide synthase (iNOS) (Vallejo et al., 2014).

### Vascular Inflammation and Atherosclerosis

Chronic vascular inflammation is at the basis of atherosclerosis, which in turn accounts for life-threatening complications of DM such as AMI or stroke. The pro-inflammatory cytokines IL-1β and IL-1α are widely expressed in human and experimental atherosclerotic lesions (Frostegård et al., 1999; Liu et al., 2014) and a large body of preclinical data reveals that IL-1β plays a major role in the progression and rupture of atherosclerotic plaques (Chi et al., 2004; Isoda et al., 2004; Merhi-Soussi et al., 2005; Chamberlain et al., 2006; Frostegård, 2013; Singla et al., 2016). Thus, a rationale is set for the use of pharmacological IL-1β blockers as tools to delay the onset and progression of atherosclerotic lesions.

One of the earliest steps of atherosclerosis is the recruitment of leukocytes by endothelial cells via the expression of adhesion molecules such as ICAM-1 and VCAM-1. The saturated fatty acid palmitate, which is linked to a higher risk of type 2 diabetes and cardiovascular disease, promotes the release of IL-1β by monocytes and their adhesion to human endothelial cells (Shikama et al., 2015). This effect can be blocked by anakinra, which prevents the induction of adhesion molecules elicited by IL-1β (Shikama et al., 2015).

An imbalanced trafficking and distribution of intracellular cholesterol has been associated with defective signaling and vascular cell dysfunction (Schreiber, 2002). In cultured vascular smooth muscle cells, serum amyloid A, which is expressed in atherosclerotic lesions, promotes cholesterol trafficking via the release of IL-1β (Pessolano et al., 2012). This effect can be inhibited by the recombinant form of IL-1Ra (Pessolano et al., 2012), and presumably, by IL-1β blocking drugs, although this point still needs to be addressed.

Moreover, the dysruption of the endoplasmic reticulum (ER) homeostasis by high glucose or other factors has been identified over the last years as an important mechanism that underlies many complications of type 2 such as endothelial dysfunction, vascular inflammation and atherosclerosis (Lenna et al., 2014). Chronic ER stress leads to the so-called unfolded protein response (UPR), which ultimately seeks to restore ER function but can also lead to cell death and apoptosis (Lenna et al., 2014). In a murine model of type 2 diabetes, the intraperitoneal injection of anakinra during 4 weeks diminished ER stress and macrophage infiltration, and improved ischemia-induced neovascularization and blood flow as compared with untreated animals (Amin et al., 2012). These effects were further accompanied by a normalization in the blood levels of cholesterol and adiponectin without affecting glycemia (Amin et al., 2012).

It is also worth noting that the pro-inflammatory signaling elicited by IL-1β in human vascular cells can be exacerbated by high glucose. In human endothelial cells, the expression of adhesion molecules such as ICAM-1 and VCAM-1 induced by IL-1β or TNF-α is augmented in the presence of extracellular high glucose (Azcutia et al., 2010). Functionally, the synergy between IL-1β and high glucose leads to an exaggerated leukocyte-endothelial adhesion *in vitro*, together with enhanced rolling flux and emigration of leukocytes in animal experimental models *in vivo* (Azcutia et al., 2010). Similarly, in human vascular smooth muscle cells IL-1β triggers the activation of pro-inflammatory pathways such as the ERK 1/2- NF-κB- inducible nitric oxide synthase (iNOS) axis (Lafuente et al., 2008). Such an effect of IL-1β is exaggerated proportionally to the concentration of extracellular glucose (Lafuente et al., 2008). As an explanation for this synergy, it has recently been shown that IL-1β permits the entry of extra glucose across the plasma membrane of human vascular smooth muscle cells (Peiró et al., 2016). Part of this excess intracellular glucose is then driven by the pentose phosphate pathway to produce NADPH that in turn fuels the pro-oxidant enzyme NADPH oxidase. The over-production of ROS seems ultimately to be responsible for the exaggerated NF-κB activation and iNOS induction triggered by IL-1β under high glucose conditions (Peiró et al., 2016). By using anakinra, not only the pro-inflammatory and pro-oxidant signaling elicited by IL-1β in vascular cells was blunted but also, and importantly, its exacerbation by extracellular high glucose (Peiró et al., 2016).

**FIGURE 1:**
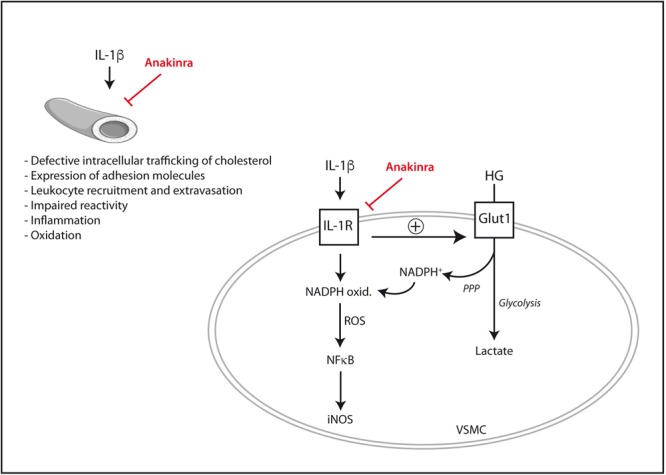
Mechanisms by which IL-1β may directly promote vascular dysfunction and their inhibition by the IL-1Ra recombinant analog anakinra. In vascular smooth muscle cells (VSMC), IL-1β synergizes with extracellular high glucose (HG) to exacerbate pro-inflammatory signaling. iNOS, inducible nitric oxide synthase; Glut1, glucose transporter 1; IL-1R, interleukin-1 receptor; NADPH ox., NADPH oxidase; PPP, pentose phosphate pathway; ROS, reactive oxygen species.

Despite the promising pre-clinical data available to date (**Figure 1**), the ability of anakinra or other IL-1β blocking drugs to prevent or retard diabetes-associated experimental atherosclerosis remains to be better established and requires additional research with *in vivo* models. The scarce data available on IL-1 blockade and atherosclerosis in patients suffering from T2DM will be presented later in this review.

### Accelerated Vascular Aging

Type 2 diabetes, together with obesity, has been shown to accelerate aging processes and, particularly, vascular aging (Minamino and Komuro, 2007; Barton, 2010). Indeed, besides presenting endothelial dysfunction and a pro-inflammatory status, animal models of obesity or diabetes exhibit core hallmarks of vascular aging and cardiovascular pathologies, such as arterial stiffness, calcification, and endothelial cell senescence (Brodsky et al., 2004; Sloboda et al., 2012). In humans, different reports have evidenced that patients suffering from type 2 diabetes display a higher propensity to calcified arteries, especially in the vasculature of lower extremities and in the coronary artery (Chen and Moe, 2003; Zhu et al., 2012), while they also exhibit enhanced arterial stiffness (Laugesen et al., 2013). The presence of senescent endothelial cells has been demonstrated in atherosclerotic lesions from aortae and coronary arteries of rat models of diabetes (Chen et al., 2002; Minamino et al., 2002). Vascular senescence is associated to a series of morphological and metabolic disturbances that result in dysfunctional cell homeostasis (Erusalimsky, 2009) The senescence-associated secretory phenotype is characterized by enhanced cytokine secretion that in turn drives local inflammation and vascular dysfunction (Erusalimsky, 2009). Overall, the occurrence of vascular premature aging in patients suffering from obesity and/or T2DM favors the development of vascular disease, markedly enhances cardiovascular risk and worsens the life expectancy of these patients. To date, there are few studies available on the impact of IL-1β on accelerated vascular aging. It is known, however, that it can promote both alkaline phosphatase expression and mineralization in cultured vascular smooth muscle cells, as two mechanisms favoring vascular calcification (Lencel et al., 2011). We have also recently observed that IL-1β can induce the accumulation of senescence-associated β-galactosidase in cultured human endothelial cells, an effect that can be dampened by anakinra (Peiró et al., unpublished results).

## Il-1β inhibition and Diabetic Cardiomyopathy

### Role of IL-1β in the Heart Failure

Heart failure (HF) is a complex clinical syndrome characterized by impaired cardiac function (left ventricular ejection fraction less than 40%), and enhanced inflammation which is associated with worsening outcomes in these patients. Although infection with microorganisms is not involved in the development of HF in most cases (sterile inflammation), inflammation has been implicated in the pathogenesis of HF. In this regard, IL-1β exerts crucial effects on most cell types involved in cardiac repair and injury, and beneficial and detrimental effects of IL-1β have been reported (Bujak and Frangogiannis, 2009). The pro-inflammatory and pro-fibrotic responses after myocardial injury serve to clear the wound and facilitate wound healing and scar formation, and an excessive inhibition of the inflammatory response can develop a defective scar (Frangogiannis et al., 2005). Also, in myocardial ischemia-reperfusion (I/R) injury, IL1β and IL1α pretreatment reduces ROS generation and a subsequent increase in glucose-6-phosphate dehydrogenase activity, which provides cytoprotective effects against oxidation (Hedayat et al., 2010). However, excessive upregulation or chronic stimulation of IL1β and IL1α have been linked to deleterious responses.

#### IL-1β and Cardiac Inflammation

**FIGURE 2:**
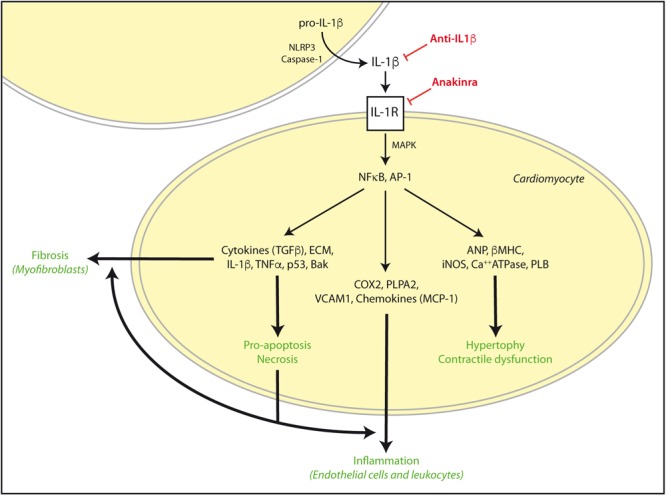
IL-1β activation in cardiac damage. After cardiac injury such as AMI or I/R, exacerbated IL-1β signaling can induce NFκB and AP-1 activation and subsequent increase of pro-hypertrophic, contractile, inflammatory, apoptosis/necrosis, and fibrotic genes for autocrine and paracrine responses. These effects are reinforced in the diabetic myocardium, and can be stimulated by TNFα and TLRs signaling. However, some drugs may specifically target IL-1β actions by NLRP3, IL-1β, and IL-1R blockade. ECM, extra cellular matrix proteins.

During an ischemic episode, myocardial contractile force diminishes, Ca^+2^ homeostasis is altered, O_2_-derived ROS are generated, NO is released, and local production of cytokines, particularly TNF-α and IL-1β is increased. IL-1β was described as an early upregulated cytokine in cardiac inflammation that becomes chronically elevated after impaired myocardial function and LV hypertrophy following AMI (Toldo et al., 2014; Fang et al., 2015). These cytokines enhance the expression of cyclooxygenase-2, and phospholipase A2, as well as vascular adhesion molecules and several chemokines. Interactions between chemokines and cell adhesion molecules activate the innate and adaptive immune system on endothelial cells of leukocytes. Then, an immediate cytokine-mediated neutrophil and mononuclear cells recruitment and extravasation in the infarcted myocardium further damages heart muscle (Herskowitz et al., 1995). In addition, IL-1β signaling, together with Toll-like receptors activation (TLR), stimulate NF-κB to increase the expression of more cytokines, chemokines, and adhesion molecules (**Figure 2**). Remarkably, NF-κB and TLRs signaling upregulate also pro-IL-1β (Fang et al., 2015). Finally, IL-1β is able to activate spleen monocytopoiesis following AMI to stimulate further monocyte production. Thus, excessive IL-1β signaling and inflammation has been linked to increased incidence of arrhythmia and other AMI-related pathologies (Fang et al., 2015).

#### IL-1β, Cardiac Hypertrophy, and Contractile Dysfunction

After a cardiac insult, IL-1β is also involved in the myocardial hypertrophic growth response by direct gene expression to compensate for environmental stresses (**Figure 2**). The expression of IL-1β is increased in pressure and volume overload-induced cardiac hypertrophy (Dai et al., 2004; Higashikuni et al., 2013), and IL-1β induced growth of isolated cardiomyocytes (McTiernan et al., 1997). Transgenic mice with constitutively cardiac-specific overexpression of IL-1β produced myocyte hypertrophy and HF (Palmer et al., 1995). This hypertrophic response was NO-independent and lead to activation of the fetal gene program by upregulation of atrial natriuretic factor and β-myosin heavy chain. In addition, IL-1β, in synergism with TNF-α, have demonstrated an exacerbation of heart contractile dysfunction. IL-1β decreased myocardial contractility by overexpressing iNOS in cardiomyocytes. Then, NO was released to promote a cardiodepressant effect by directly blocking the mitochondrial activity and reducing energy depletion (Stein et al., 1996). This effect of IL-1β was enforced by amelioration of calcium regulatory genes such as sarcoplasmic reticulum Ca^2+^-ATPase, phospholamban, and voltage dependent calcium channel, and subsequent decrease of basal and stimulated contractility (Oddis and Finkel, 1995; Tatsumi et al., 2000).

#### IL-1β, Cardiac Necrosis/Apoptosis, and Autophagy

The uncontrolled inflammation in the injured heart, as in AMI, induces cellular apoptosis and progressive cardiac dysfunction. The progression from cardiac injury to symptomatic HF is mainly due to a loss of functional cardiac myocytes through necrosis, autophagy/mitophagy, intrinsic and extrinsic apoptosis (Gordon et al., 2011). Autophagy and mitophagy have been recently characterized as essential cellular processes in the heart, but whether these functions as pro-death or pro-survival programs during disease conditions is still not completely understood. The link between autophagy, mitochondria, and inflammation in the heart has been established (Oka et al., 2012). In particular, in diabetes, mitochondrial DNA, a by-product of insufficient mitophagy, induced sterile inflammation and subsequent cardiac dysfunction through the TLR9 pathway, and increasing IL-1β, IL-6, and infiltration of CD68^+^ macrophages.

Nevertheless, myocyte extrinsic apoptosis is the most significant mechanism of cell loss and the end point of pathological remodeling induced by cardiac injury, enhanced autonomic activity, and cytokine secretion (**Figure 2**). In AMI, the acute releasing of IL-1β and TNFα can regulate the survival or apoptosis of myocytes in infarcted zone. They also produce a negative inotropic effect as an adaptive response to delimit the injury and decrease myocardial energy demand (Schulz et al., 1995). *In vitro*, in cultured cardiac myocytes, IL-1β induced programmed cell death through a cGMP-independent NOS induction, and by generation of ROS, activation of caspases, and alteration of the cellular Bak/Bcl-xL ratio (Abbate et al., 2008). On the other hand, the presence of necrosis has been demonstrated in most cardiac injuries (Shinde and Frangogiannis, 2014). Necrotic cells release danger signals, activating innate immune pathways and triggering an intense inflammatory response. Stimulation of TLR signaling and complement activation prompted NF-κB and related upregulation of proinflammatory cytokines (i.e., IL-1β, IL-1α, and TNFα) and chemokines (i.e., MCP-1 and CCL2). Interestingly, some NF-κB target genes such as TNFα, and p53 directly induced also intrinsic apoptosis (Shakhov et al., 1990).

#### IL-1 and Cardiac Fibrosis

The adult mammalian myocardium contains abundant fibroblasts entrapped within the interstitial and perivascular extracellular matrix (ECM). After a massive sudden loss of cardiomyocytes following AMI, the limited regenerative capacity of the myocardium overcomes, resulting in the formation of a collagen-based scar (Prabhu and Frangogiannis, 2016). In the early stages of infarct healing, fibroblasts become pro-inflammatory cells, activating the inflammasome and producing cytokines, chemokines and proteases. NLRP3 inflammasome is predominantly upregulated in the cardiac fibroblasts of the ischemic myocardium in animal models with AMI (Fang et al., 2015). Then, pro-inflammatory cytokines, such as Interleukin-1, delay myofibroblast transformation, until the wound is cleared by infiltrated leukocytes from dead cells and matrix debris. Suppression of the inflammatory response triggers activation of reparative cells. Thus, fibroblasts migrate, proliferate, undergo myofibroblast transdifferentiation, and deposit large amounts of ECM proteins to maintain the structural integrity of the infarcted myocardium. In particular, IL-1 signaling regulates reparative processes by modulating gene expression of growth factors and ECM proteins (i.e., procollagen α1-IV, α2-IV, and fibronectin) in fibroblasts and smooth muscle cells, and by altering the Matrix Metalloproteinase (MMP)/Tissue Inhibitor of Metalloproteinases (TIMP) balance (Prabhu and Frangogiannis, 2016) (**Figure 2**). IL-1 increased MMP-1, MMP-3, MMP-7, MMP-9, and MMP-13, and TIMP-1 and TIMP-2, via AP-1 and NF-κB activation (Siwik and Colucci, 2004). Again, the myocardial matrix also elicits TLRs and IL-1 signaling, which in turn stimulates NF-κB. The renin–angiotensin–aldosterone system and members of the TGF-β family play also a pivotal role in activation of myofibroblasts (Siwik et al., 2000). Formation of a mature cross-linked scar is associated with clearance of fibroblasts following inhibitory signals to restrain the fibrotic response (Shinde and Frangogiannis, 2014). However, excessive fibrosis has been linked to increased incidence of arrhythmia and other AMI-related pathologies (Xie et al., 2004). In this sense, significant upregulation of IL-1 can extend to non-infarcted areas and promote a second phase of elevated levels of cytokines, leading to interstitial fibrosis in the non-infarcted myocardium, and enhancing cardiac dysfunction (Shinde and Frangogiannis, 2014).

### Blockade of IL-1β in Experimental HF

Several reports have demonstrated the benefit of blocking IL-1β in experimental models of HF. Early inhibition of IL-1β signaling is more likely to inhibit the inflammatory cascade, whereas late inhibition may predominantly abrogate the direct actions of IL-1β on fibroblasts (Bujak et al., 2008). In rodents, by using genetically engineered antibody to IL-1β, cardiac enlargement and dysfunction following AMI were reduced without affecting the infarct size (Abbate et al., 2010). By administration of a recombinant IL-1Ra in T1DM-induced diabetic cardiomyopathy, the IRAK2/CHOP-dependent apoptosis was attenuated, without affecting fasting blood glucose concentration (Liu et al., 2015). Interestingly, in T1DM-induced diabetic cardiomyopathy, administration of an anti-TNF-α monoclonal antibody lessen cardiac TNF-α and IL-1β expression in correlation with cardiac collagen-I and -III content, and improvement of left-ventricle function (Westermann et al., 2007).

Other strategies to control IL-1β activity in the context of cardiomyopathy address the kallikrein–kinin system and the NLRP3/caspase-1/TLR pathway. Transgenic activation of kallikrein-1 ameliorated intramyocardial inflammation through reduction of adhesion molecules, IL-1β and TNF-α, and leukocyte infiltration, as well as endothelium dysfunction and oxidative stress in T1DM-induced diabetic cardiomyopathy (Tschöpe et al., 2005). Moreover, NLRP3-deficient mice subjected to I/R exhibited a marked improvement of cardiac function and reduction of hypoxic damage (Sandanger et al., 2013). NLRP3 gene silencing ameliorated pyroptosis and ROS release under high glucose in cardiomyocytes. ROS inhibition decreased also NF-κB and mature IL-1β (Luo et al., 2014a). Finally, NLRP3 and high-mobility group box-1 (HMGB1) knockdowns reduced cardiac hypertrophy and fibrosis, and restored cardiac function (Fuentes-Antras et al., 2014). Ablation of TLR4 successfully reverted architectural aberrations and restored cardiac dysfunction in T1DM mice. Thus, the role of HMGB1 as TLR4 ligand and upstream inducer of NF-κB and NLRP3 may shape a key pathogenic axis in diabetic cardiomyopathy, suggesting their potential as novel anti-inflammatory approaches. In this context, other ligands for TLR (TLR2 and TLR4) such as biglycan could also play a key role in amplifying fibrotic responses after cardiac injury (Beetz et al., 2016).

In this regard, statins as rosuvastatin have also exhibited beneficial proprieties against diabetic cardiomyopathy through inhibition of NLRP3 inflammasome and mature IL-1β, via suppression of the MAPKs activation (Luo et al., 2014b). Similar effects for pravastatin were observed also in obese T2DM rats with AMI (Li et al., 2006). Moreover, administration of the PDE-5 inhibitor tadalafil reduced circulating IL-1β and TNF-α, and associated chemokines RANTES, MIP-1β and MCP-1, after I/R induction in T2DM mice. In parallel, tadalafil upregulated the anti-inflammatory cytokine IL-10 and improved fasting glucose, whereas decreased infarct size (Varma et al., 2012). In addition, a glucocorticoid (methylprednisolone) treatment reduced expression of TLR4/NF-κB signaling and IL-1β, IL-6, TNF-α, and ICAM-1 on T1DM-induced diabetic cardiomyopathy with I/R injury (Hu et al., 2011). More importantly, the antiapoptotic property of anakinra has been demonstrated in models of I/R injury and AMI. This action was due to a decreased expression of pro-apoptotic mediators Bax, Bak, and caspase-1 and -3, which promote a reduction of infarct size and favorable ventricular remodeling (Abbate et al., 2008). Also, in T1DM mice, released IL-1β from cardiac macrophages stimulated with TLR2 and NLRP3 agonists, induced a decrease in potassium current and an increase in calcium sparks in cardiomyocytes, and subsequent cardiac arrhythmia. Interestingly, inhibition of IL-1β signaling by either anakinra or NLRP3 inhibitor (MCC-950) reduced these effects (Monnerat et al., 2016).

## Clinical Trials Assessing Il-1β Inhibition and Cardiovascular Complications in Patients With DM

Although pharmacological inhibitors of the IL-1 pathway were first developed to control classical autoinflammatory diseases such as rheumatoid arthritis, the increasing evidence about a key role for this pathway in the pathophysiology of T1DM and T2DM (see reviews by Dinarello, 2014; Ballak et al., 2015; Herder et al., 2015; Pollack et al., 2016) provided a rationale for assessing their therapeutic value in the context of DM. Moreover, a growing bulk of pre-clinical data highlight the potential relevance of IL-1β in the development of atherosclerosis and cardiovascular dysfunction. Despite this, the number of studies conducted in humans to assess the benefit of IL-1β inhibition on cardiovascular outcomes in the context of DM still remains very limited today.

Interestingly, the success of the first clinical trial reported using a IL blocker, i.e., anakinra, in T2DM (Larsen et al., 2007; ClinicalTrials.gov identifier: NCT00303394) reinforced the proposal that the pharmacological control of the imbalance of the IL-1:IL-1Ra ratio could be a relevant approach for the treatment of this metabolic autoinflammatory disease. In such study, 70 adult patients were treated with a 100 mg daily dose during 13 weeks. An improvement of several markers of glucose metabolism, such as reduction in HbA1c and an increased C-peptide secretion were obtained, together with the reduction of systemic inflammatory markers, such as CRP and IL-6 levels (Larsen et al., 2007). Moreover, a 39-week follow-up of the same study after treatment withdrawal, showed not only a maintained reduction in inflammatory markers but also a persistent improvement in β-cell function, as expressed by the lower proinsulin/insulin ratio in the formerly anakinra-treated patients as compared with those in the placebo group (Larsen et al., 2009). However, this study did not assess any cardiovascular outcome in the patients enrolled.

### Cardiovascular Outcomes

Although the results obtained with anakinra were encouraging, still there are characteristics that make the drug unsuitable for long-term treatment of T2DM, such as a short half-life (anakinra should be administered on a daily basis to maintain adequate suppression of IL-1β) or injection-site reactions, in addition to being an expensive option. It is also worth to note that an anti-IL-1 strategy that saves IL-1α activity could offer safety benefits. In fact, genome-wide association studies recently suggested that long-term dual IL-1α/β inhibition could increase cardiovascular risk mediated, at least in part, through an increase in proatherogenic lipid concentrations (The Interleukin 1 Genetics Consortium, 2015). Therefore, reducing specifically IL-1β activity with longer-lasting agents would provide a therapeutic improvement in terms of sustained inhibition of IL-1β. In this context, the monoclonal antibody canakinumab achieves IL-1β inhibition, while preserving IL-1α activity. Also, the significantly longer half-life of canakinumab (Chakraborty et al., 2012) makes the drug more suitable for assessing outcomes that may require longer treatment times, such as cardiovascular ones.

In fact, canakinumab is currently being tested in the Canakinumab Anti-inflammatory Thrombosis Outcomes Study (CANTOS), which is the largest trial in progress for any anti-cytokine drug (ClinicalTrials.gov identifier: NCT01327846). The objective is to test whether canakinumab will reduce cardiovascular events in high-risk patients with T2DM who have high CRP levels (hsCRP > 2 mg/L) despite receiving optimal statin therapy. The trial is examining 17,200 individuals using three doses of the antibody in a placebo-controlled, randomized design in 146 centers. Although the primary outcome is based on the hypothesis that blocking IL-1β activity in these patients may also reduce the incidence in myocardial infarction and stroke, secondary end points include prevention and improvement of diabetes. CANTOS is an event driven trial due to be completed in 2017, when approximately 1400 cases of myocardial infarction, stroke, or cardiovascular death will have accrued (Ridker et al., 2011).

A preliminary proof-of-concept study has been already executed within the CANTOS trial; thus, 556 well-controlled type 2 diabetic subjects (baseline HbA1c of 7.4%) with high cardiovascular risk have been randomly allocated to receive monthly subcutaneous canakinumab at doses of 5, 15, 50, or 150 mg or placebo and followed over 4 months. Together with an important dose-dependent reduction in inflammatory markers (CRP and IL-6), there was a modest non-significant improvement in glucose control parameters (HbA1c, glycemia, and insulinemia), while lipoprotein levels were unchanged (Ridker et al., 2012). In terms of cardiovascular outcomes, there is great expectation for the results of the trial. If positive, CANTOS would reinforce the inflammatory hypothesis of atherothrombosis and support a cytokine-based therapy for the secondary prevention of cardiovascular disease in T2DM.

Apart from the expected results from CANTOS, there are very few studies available on vascular outcomes in patients with DM and most of them show no clear cardiovascular improvement upon IL-1β blockade. A smaller trial enrolled 189 patients with atherosclerosis in the carotid artery and/or the aorta and either T2DM or impaired glucose tolerance (ClinicalTrials.gov identifier: NCT00995930). The subcutaneous administration of canakinumab (150 mg) monthly for 12 months did not significantly affect vascular structure in terms of carotid intima thickness, or vascular function in terms of aortic distensibility measured by pulsed wave velocity (Choudhury et al., 2016). Canakinumab showed an anti-inflammatory effect by reducing the circulating levels of IL-6 and hs-CRP, but had no effect on metabolic indicators, like fasting glucose, HbA1c, or insulin sensitivity (Choudhury et al., 2016). Although no cardiovascular benefit was found, the results were not considered conclusive due to some limitations, including the short duration of the study to assess structural changes. Also, the lack of stratification of the patients enrolled based on their initial inflammatory status did not permit to identify those patients that would potentially benefit more from IL-1β inhibition. Finally, the dose administrated was sufficient to lower inflammatory markers but perhaps was not high enough to generate a maximal effect on atherosclerotic lesions.

Another prospective non-controlled pilot study was performed with only six patients with proliferative diabetic retinopathy, secondary to T1DM or T2DM, and receiving subcutaneous canakinumab (150 mg) every 8 weeks (ClinicalTrials.gov identifier: NCT01589029). No regression of retinal neovascularization was reported after 24 weeks of follow-up, although a positive effect on macular edema was observed (Stahel et al., 2016). Again, the dose of canakinumab may have been insufficient, with higher doses of 300 mg every 4 weeks being currently approved for uses such as systemic juvenile idiopathic arthritis.

A possible limitation for these studies, in addition to sample size or dosage, can be that the patients enrolled already exhibited advanced stage cardiovascular disease. Thus, parallel studies performed in diabetic patients without evidence of advanced vascular lesions would help shedding light on the capacity of canakinumab or other IL-1β blocking drugs to prevent or retard the onset of atherosclerosis or other vascular alterations in the context of DM.

### Safety

Canakinumab was safe and well-tolerated in the above-mentioned cardiovascular trials, with no significant differences in adverse effects between treated and control groups (Choudhury et al., 2016; Stahel et al., 2016). The upcoming results of CANTOS will help shedding more light on the tolerability of long-term treatments with canakinumab.

In the context of T2DM, other studies not specifically designed to assess cardiovascular outcomes, have already provided information on the tolerability of canakinumab. A recent study pooled the data from trials that used different doses of the drug (Howard et al., 2014). These trials enrolled a total of 1026 patients with different routes of administration, treatment regimes and follow-up time (ClinicalTrials.gov identifiers: NCT00900146 and NCT00605475; Ridker et al., 2012; Rissanen et al., 2012; Hensen et al., 2013; Noe et al., 2014). The global analysis of the three trials demonstrated that canakinumab was safe and well tolerated over a treatment period up to 1.4 years at the four pooled doses evaluated, which was in agreement with safety findings reported in the individual studies (Howard et al., 2014). No significant differences were evidenced in terms of adverse effects, discontinuations or deaths between treatment and placebo (Howard et al., 2014). Overall, canakinumab seems safe and well tolerated by patients with T2DM. However, additional trials with longer follow-up times are still needed to assess whether the drug will be suitable for longer-term treatment of a chronic conditions such as T2DM.

## Concluding Remarks

The growing body of evidence pointing at IL-1β as a key player in the development of DM and its cardiovascular complications provides nowadays a solid rationale for IL-1 blockade as a potential pharmacological approach to treat the disease. While IL-1 inhibition seems to be rather promising in controlling the pro-inflammatory status and even in ameliorating glucose homeostasis in the context of DM, evidence is lacking on its potential benefits on the cardiovascular complications of the disease.

Most of the positive results obtained to date in terms of cardiovascular improvement arise from pre-clinical data mostly performed through *ex vivo* or *in vitro* approaches. Clearly, further experimental studies using *in vivo* experimental models are required to better understand the pharmacological effects and mechanisms of actions of IL-1β blockers on the cardiovascular system. This will help identifying which conditions may benefit more from IL-1β blockade and predicting potential adverse effects.

At present, the knowledge derived from studies in humans remains very limited. Despite promising results, additional studies need to be performed to better assess the tolerability, and, importantly, the long-term safety of the drugs that may achieve long-lasting IL-1β blockade. In terms of clinical efficacy, more trials are needed with an accurate patient selection based on the stage and complications of the disease. Studies such as the CANTOS trial are soon expected to shed light on the impact of long-lasting IL-1β inhibitors on the cardiovascular complications of DM. This and forthcoming studies, that may include novel pharmacological approaches such as therapeutic vaccination, will allow answering whether or not IL-1β is indeed a valuable therapeutic target to reduce the burden of cardiovascular diabetic complications.

## Author Contributions

CP, OL, RC, and CS-F designed, wrote, reviewed, and approved the manuscript.

## Conflict of Interest Statement

The authors declare that the research was conducted in the absence of any commercial or financial relationships that could be construed as a potential conflict of interest.
